# Two *LEAFY* homologs *ILFY1* and *ILFY2* control reproductive and vegetative developments in *Isoetes* L.

**DOI:** 10.1038/s41598-017-00297-3

**Published:** 2017-03-22

**Authors:** Tao Yang, Ming-fang Du, You-hao Guo, Xing Liu

**Affiliations:** Laboratory of Plant Systematics and Evolutionary Biology, College of Life Science, Wuhan University, Wuhan, Hubei China

## Abstract

*LEAFY* (*LFY*) is a plant-specific transcription factor, which is found in algae and all land plants. *LFY* homologs exert ancestral roles in regulating cell division and obtain novel functions to control floral identity. *Isoetes* L. is an ancient genus of heterosporous lycophytes. However, characters about *LFY* homologs in lycophytes remain poorly investigated. In this study, two *LFY* homologs, *ILFY1* and *ILFY2*, were cloned from five *Isoetes* species, including *I. hypsophila*, *I. yunguiensis*, *I. sinensis*, *I. orientalis*, and *I. taiwanensis*. The full length of *ILFY1* was 1449–1456 bp with an open reading frame (ORF) of 927–936 bp. The full length of *ILFY2* was 1768 bp with ORF of 726 bp. Phylogenetic tree revealed that *ILFY1* and *ILFY2* were separated into two clades, and *I. hypsophila* were separated with the others. Expression analysis demonstrated that *IsLFY1* and *IsLFY2* for *I. sinensis* did not show functional diversity. The two transcripts were similarly accumulated in both vegetative and reproductive tissues and highly expressed in juvenile tissues. In addition, the *IsLFY1* and *IsLFY2* transgenic *Arabidopsis* similarly did not promote precocious flowering, and they were inactive to rescue *lfy* mutants. The results facilitate general understandings about the characteristics of *LFY* in *Isoetes* and evolutionary process.

## Introduction

Flowering is a common and critical event during the life cycle of angiosperm plants. During vegetative phase, apical meristems give rise to leaves and lateral shoots. Then plants switch from vegetative into reproductive development, and flowers are initiated which is controlled by floral meristem identity genes, including *APETALA1*(*AP1*) and *LEAFY* (*LFY*). *LFY* is a plant-specific transcript factor, which is found in algae and all land plants from mosses to angiosperms^1–3^. *LFY* in *Arabidopsis thaliana* is a master regulator for flower initiation and determining floral fate in lateral meristems^4, 5^. It regulates downstream genes to produce floral tissues according to the ABC model for floral organ identity^6^. Increasing copy number of the endogenous *LFY* promotes early flowering and terminates inflorescence apices with solitary flowers^7, 8^.

Roles of the *LFY* homologs have been investigated in numerous species^4, 9–11^. *FLORICAULA* (*FLO*) in *Antirrhinum majus* is expressed in bract primordia, sepal, petal, and carpel primordia^10^. However, *RLF*, a *LFY* homology in *Oryza sativa*, controls the branching of inflorescence^12^. The function of *RLF* for floral identity is partially lost, which has been replaced by another factors^13^. The *unifoliata* pea mutants alter in both floral development and leaf morphology^14^. It is generally considered that *LFY* homologs possess two functions in angiosperms, including conferring floral identity and promoting meristem growth^15, 16^. However, the two functions may be all prominent (such as in pea, maize and tobacco) or one function may be reduced and the other is obvious (such as in *A. thaliana* and *O. sativa*)^4, 9, 14, 16^. Another related paralog, *NEEDLY* is identified in gymnosperms and lost in angiosperms^17^. But only *LFY* in gymnosperms is orthologous with *LFY* in angiosperms. The *LFY* and *NEEDLY* paralogs in gymnosperms are specifically expressed in both vegetative and reproductive meristems^18, 19^. The *LFY* paralog provides more activity to rescue *Arabidopsis lfy* mutant phenotypes than *NEEDLY* ^1, 18^.

In addition, *LFY* homologs are also present in free-sporing land plants, including lycophytes, ferns or their allies and bryophytes^1, 2, 20^. Expressions of *CrLFY1* and *CrLFY2* in *Ceratopteris richardii* are prominent in shoot tips and circinate reproductive leaves^20^. But *CrLFY2* only has some ability to rescue severe *Arabidopsis lfy* mutants, which is less than *LFY* homologs in gymnosperms^1^. Two *PpLFY* paralogs in the moss *Physcomitrella patens* are markedly expressed in gametophyte and sporophyte^21^. The PpLFY proteins play critical roles in controlling first zygotic cell division^21^. Whereas they are inactive to complement *Arabidopsis lfy* mutants^1^. Taken together, these observations imply that translation from the vegetative to reproductive development and directed induction of the floral homeotic MADS-box genes by *LFY* are established after the divergence of mosses and before the divergence of vascular plant lineage^19, 20^. *LFY* homologs probably regulate cell division, expansion and arrangement in free-sporing land plants, and they control both floral identity and cell division in seed plants^16, 22^. Additionally, *LFY* homolog is not specific to land plants and it is also found in algae, indicating ancestral roles predating land plants^2, 3^.

*Isoetes* L. is an ancient genus of heterosporous lycopsid^23, 24^. Phylogenetic analyses show that *Isoetes* is one of the earliest basal vascular plants, which can date back to the Devonian^25, 26^. *Isoetes* has approximately 200 species recognized by a strong reduced plant body^23, 25, 27^. Furthermore, this genus is the only survival of ancient taxa as the closest relatives of the famous tree lycopods^23–25^. The plants have fleshy corms with a range of spirally arranged microphylls and multiple dichotomizing roots along with median furrows of the corm^28^. Microsporangia and megasporangia are embedded in the bottom of fertile leaves. With the corms growing in girth, the leaves and sporangia are becoming mature from inside to outside^29^. It is prevalent to identify species and analyze phylogenetic relationships using second intron of *LFY* homologs^30–32^. Although *Isoetes* possesses an important position in phylogenetic evolution, characters and functions of *LFY* homologs for *Isoetes* (referred as *ILFY*) are still ambiguous.

In this present study, we cloned two *ILFY* paralogs, including *ILFY1* and *ILFY2*. Totally, we identified the two genes in five *Isoetes* species, including *I. hypsophila*, *I. yunguiensis*, *I. sinensis*, *I. orientalis*, and *I. taiwanensis*. Roles of the two *ILFY* paralogs in *I. sinensis* (*IsLFY1* and *IsLFY2*) were further investigated using the quantitative real-time PCR (qRT-PCR) and *in situ* hybridization assays. Moreover, we created transgenic *Arabidopsis* plants, which were constitutively expressed *IsLFY1* and *IsLFY2* under the control of the cauliflower mosaic virus 35S promoter, respectively. This comprehensive study will facilitate our understandings about the roles of *LFY* homologs in *Isoetes* and evolutionary process.

## Results

### Cloning of *ILFY1* and *ILFY2* sequences

**Figure 1:**
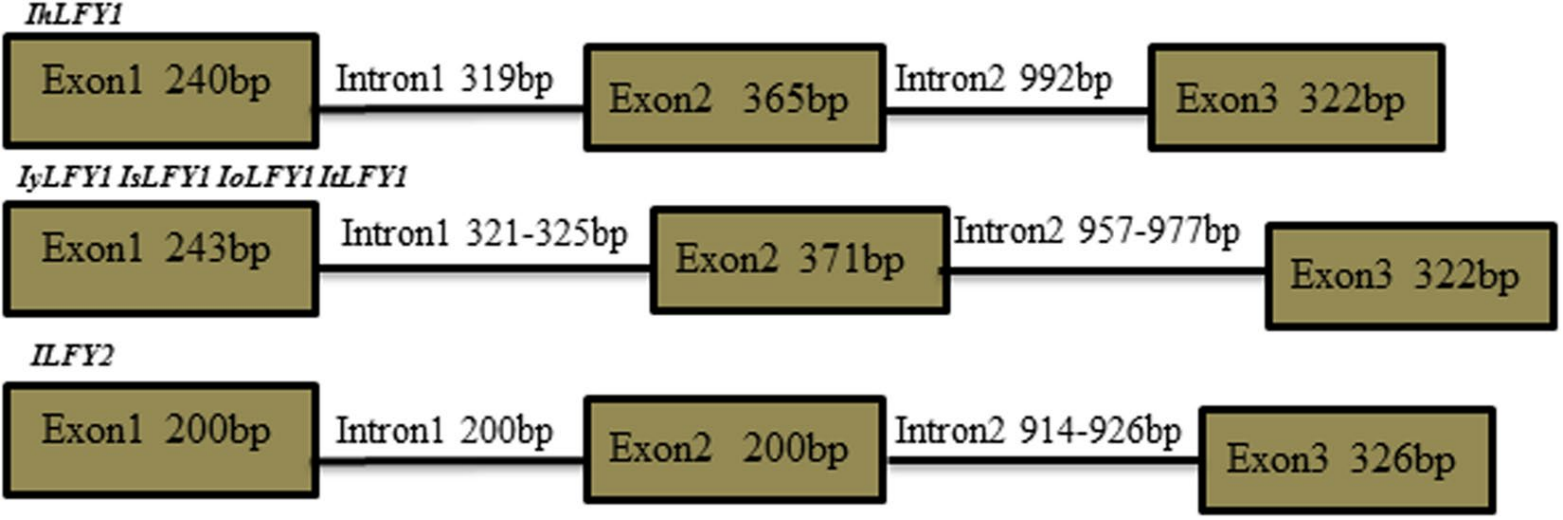
Gene structures of *ILFY1* and *ILFY2* homologs. Three exons and two introns were shown as green boxes and black lines, respectively. The *ILFY1* and *ILFY2* sequences were identical for *I. yunguiensis*, *I. sinensis*, *I. orientalis*, and *I. taiwanensis*. The two introns were variable in the five species.

The full-length of *ILFY* genes were cloned from five *Isoetes* species, including *I. hypsophila*, *I. yunguiensis*, *I. sinensis*, *I. orientalis*, and *I. taiwanensis*. In total, we isolated two *ILFY* paralogs, including *ILFY1* and *ILFY2*, based on the 5′ and 3′ rapid amplifications of cDNA ends (RACE) systems. The full length of *ILFY1* was 1449–1456 bp with an open reading frame (ORF) of 927–936 bp (Fig. 1). The full length of *ILFY2* was 1768 bp with ORF of 726 bp. More detailed information about *ILFY1* and *ILFY2* was list in Table S1, including the full length cDNA, 5′UTR, open reading fragment (ORF), 3′UTR of the two homologs in the five species. The ORF sequences of *ILFY1* and *ILFY2* were identical for *I. yunguiensis*, *I. sinensis*, *I. orientalis*, and *I. taiwanensis*. *ILFY1* for the four species occupied 94.86% identity with *IhLFY1*, and *ILFY2* shared 98.68% identity with *IhLFY2*. Furthermore, The ORF fragments for *ILFY1* and *ILFY2* for *I. yunguiensis*, *I. sinensis*, *I. orientalis*, and *I. taiwanensis* shared 63.41% identity. The ORF fragments for *IhLFY1* and *IhLFY2* displayed 62.57% identity. The corresponding sequences have been submitted into the GenBank database with the accession numbers from KX229755 to KX229764.

We also amplified and sequenced genomic *ILFY1* and *ILFY2* sequences in the five *Isoetes* species. Comparison analysis revealed that both *ILFY1* and *ILFY2* in the five *Isoetes* species contained three exons and two introns (Fig. 1). The length of first intron for *ILFY1* was 319–325 bp and second intron was 957–992 bp in length. The first intron for *ILFY2* was 200 bp in length and second intron was 914–926 bp in length.

### Protein comparison and phylogenetic tree

**Figure 2:**
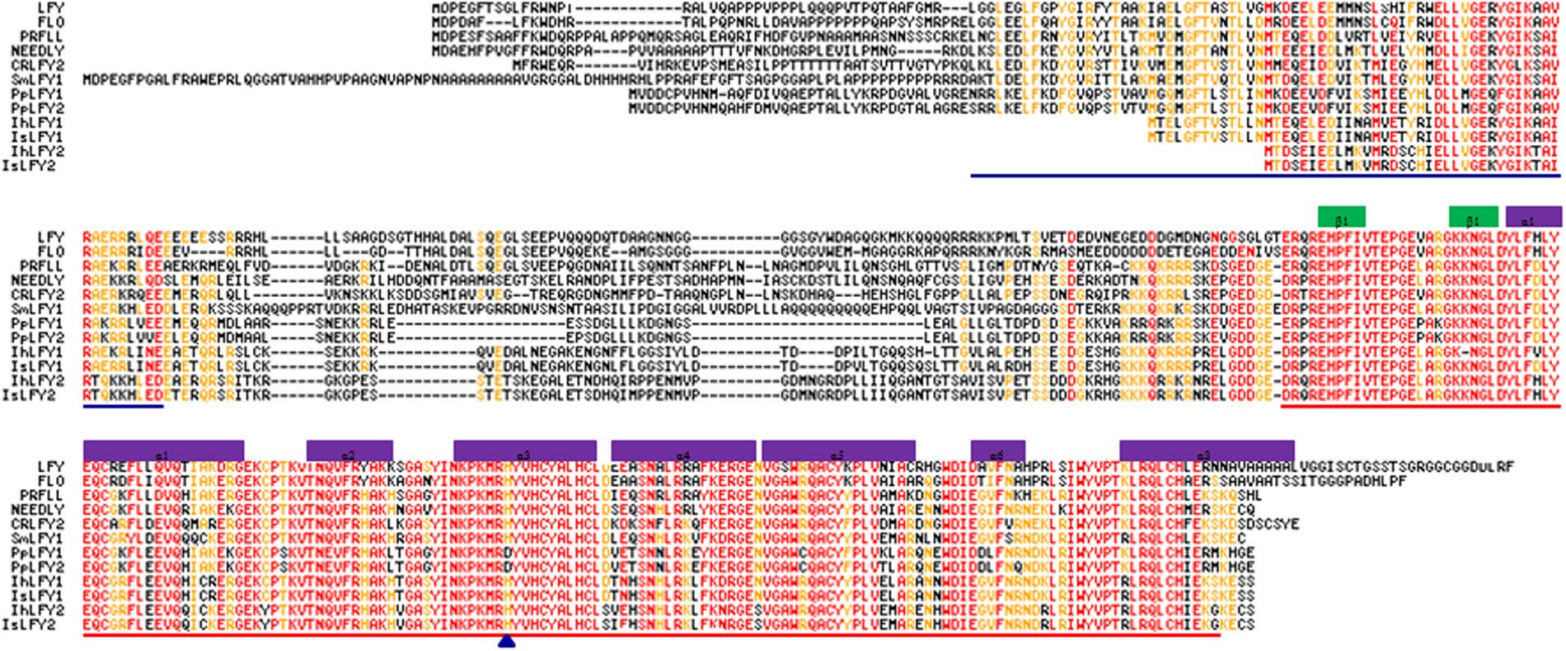
Sequences comparison of deduced LFY proteins. Deduced amino acid sequences of IhLFY1 and IhLFY2 for *I. hypsophila*, as well as IsLFY1 and IsLFY2 for *I. sinensis* were compared with LFY homologs, including LFY (*Arabidopsis thaliana*, AAA32826), FLO (*Antirrhinum majus*, AAA62574.1), PRFLL (*Pinus radiata*, O04116), NEEDLY (*Pinus radiata*, AAB68601.1), CRLFY2 (*Ceratopteris richardii*, BAB41070.2), SmLFY1 (*Selaginella moellendorffii*, XP_002978027), PpLFY1 (*Physcomitrella patens*, BAB60676.1), and PpLFY2 (*Physcomitrella patens*, BAB60677.1). Conservatively substituted and identical residues were depicted on a red background, and slightly conserved residues were on an orange background. N-terminal and C-terminal domains were overlined in blue and red, respectively. Second structures of the C-terminal domains including two β-sheet and seven α-helix, were indicated. Gaps were shown as dashes to maximize the alignments. The blue triangle represented a conspicuously different amino acid between *P. patens* and the other species.

The predicted ILFY1 and ILFY2 amino acid sequences were aligned with a diverse of LFY homologs downloaded from the NCBI database. The comparison analysis revealed that the N-terminal regions were partially conserved and C-terminal regions were highly conserved in evolutionary process (Fig. 2). Moreover, the clearly conserved C-terminal domains were comprised of two β-sheet and seven α-helix structures. Identical levels of amino acid sequences were shown in Table S2 through comparing deduced proteins of IhLFY1, IhLFY2, IsLFY1,and IsLFY2 with LFY in *A. thaliana*, PRLFY1 and NEEDLY in *Pinus radiata*, CRLFY2 in *Ceratopteris richardii*, SmLFY1 in *Selaginella moellendorffii*, and PpILFY1 in *P. patens*, respectively. A conserved histidine residue (His) is substituted by Asp in the C regions for PpILFY1 and PpILFY2 in *P. patens* ^1^. Nonetheless, The ILFY1 and ILFY2 proteins in the C regions consistently contained the conserved His residue, which was similar with that in numerous land plants^1^.

**Figure 3:**
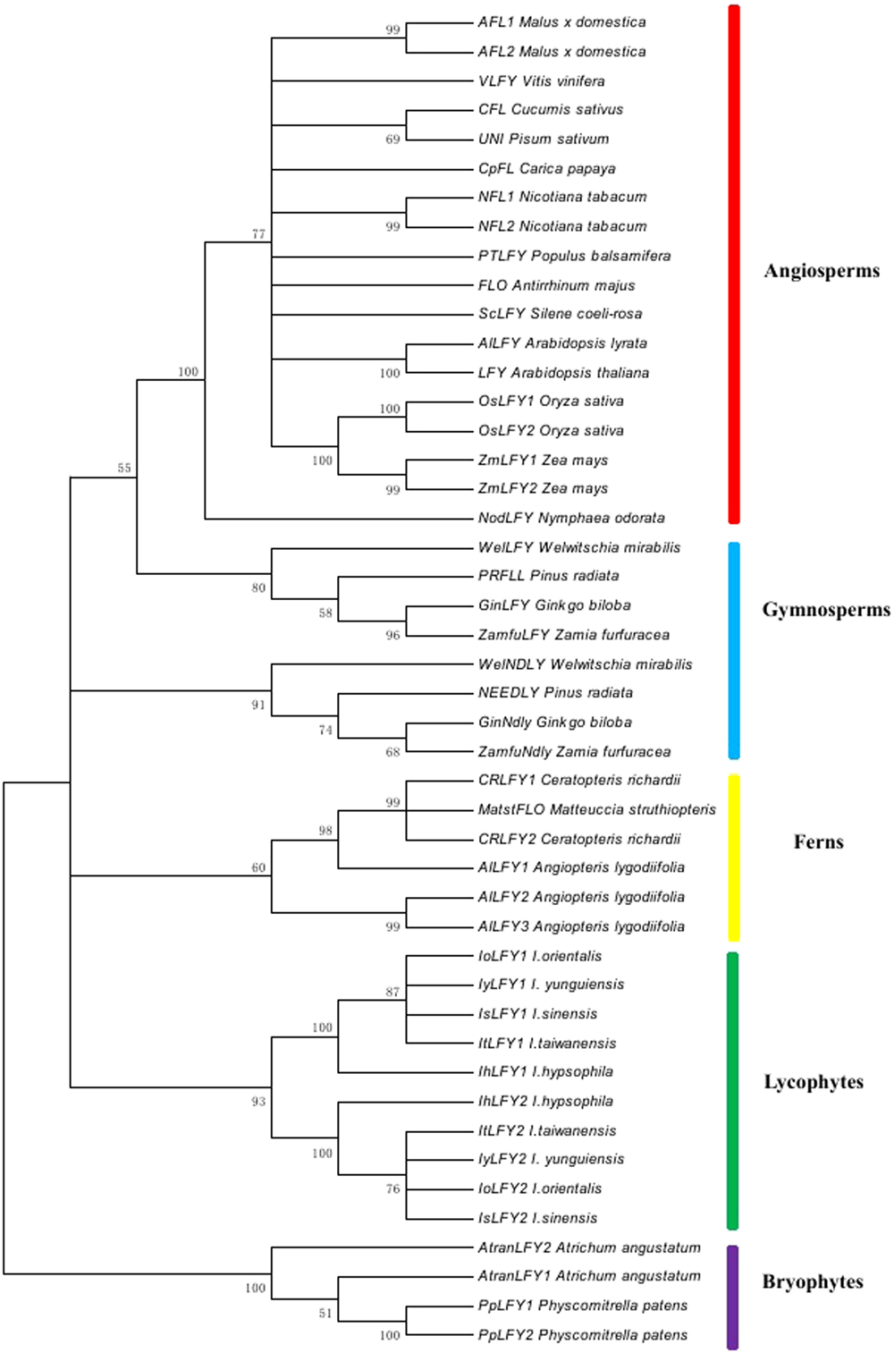
Phylogenetic tree of LFY homologs. The phylogenetic tree was constructed using the amino acid sequences of LFY homologs. Numbers above the branches represent bootstrap value, and bootstrap less than 50% was removed. Accession numbers for the LFY homologs in the dataset were listed in Table S5.

To determine the phylogenetic relationships of ILFY1 and ILFY2, we constructed a phylogenetic tree of LFY amino acid sequences using the maximum likelihood method (Fig. 3). The phylogenetic tree showed that topology of LFY homologs was concordant with the species topology^33, 34^. The ILFY1 and ILFY2 paralogs in the five *Isoetes* species were separated into two clades. Moreover, the two paralogs in *I. hypsophila* were separated with the other four species. In addition, we also used nucleotide sequences of the conserved N-terminal and C-terminal regions to reveal the phylogenetic relationships of *ILFY1* and *ILFY2*. The phylogenetic tree was similar with the phylogenetic tree based on the amino acid sequences (Figure S1). The *ILFY1* and *ILFY2* paralogs in the five *Isoetes* species were also separated into two clades and the two paralogs in *I. hypsophila* were separated with the other four species.

### qRT-PCR

**Figure 4:**
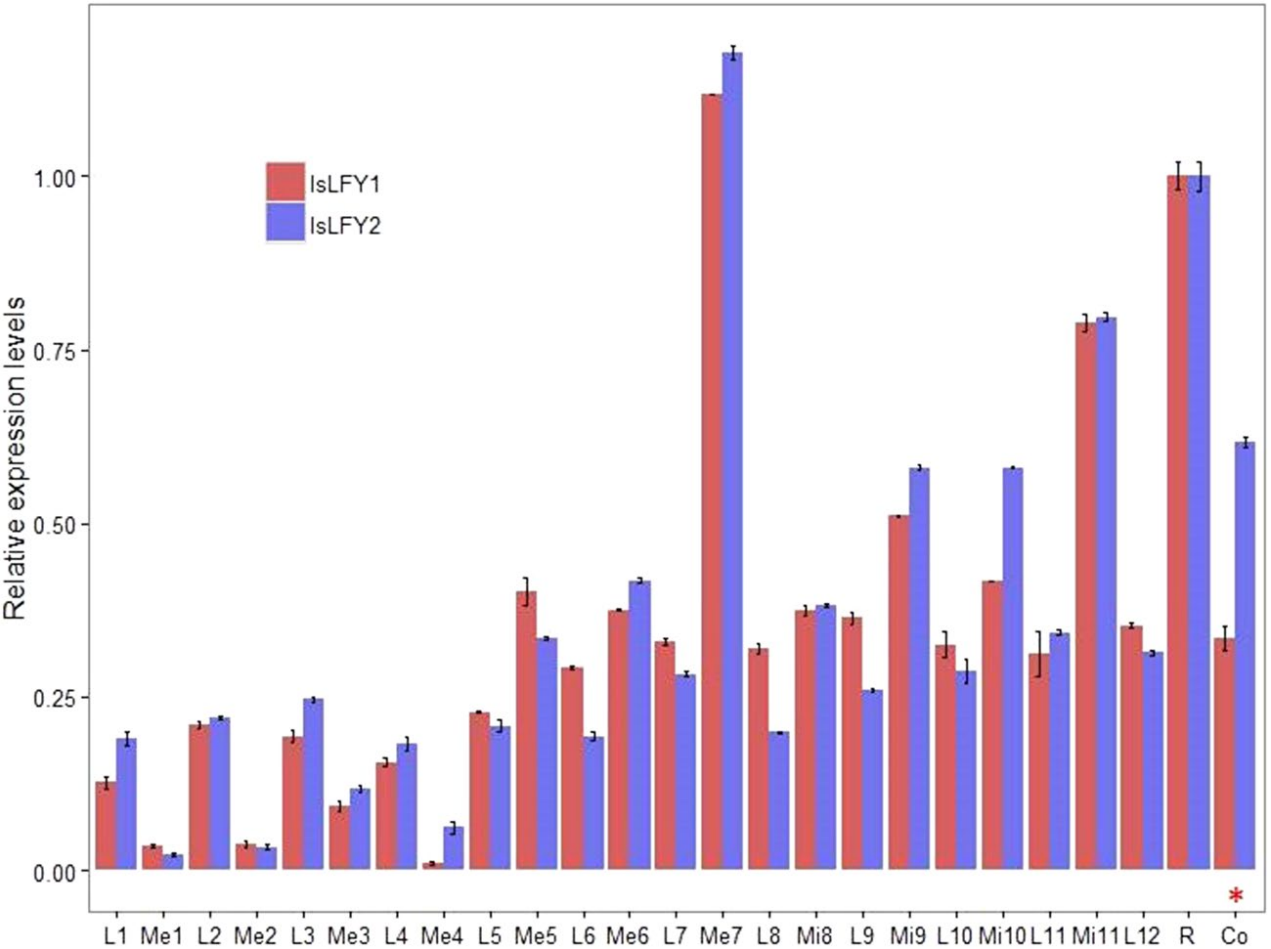
Expression patterns of *IsLFY1* and *IsLFY2* using quantitative real-time PCR assays. The abbreviations L, Me, Mi, R, Co represent leaves, megasporangia, microsporangia, roots, and corms, respectively. The megasporangia were in the bottom of fertile leaves from 1th to 7th whorls, and microsporangia were from 8th to 11th whorls. The immature leaves were in the 12th whorl. Values represent the means ± SE. Asterisk indicated that the expression levels of *IsLFY1* and *IsLFY2* were significantly different with the *P* value less than 0.01.

The expression levels of *IsLFY1* and *IsLFY2* were investigated using qRT-PCR approach, respectively. The materials included roots, corms, and each whorl of leaves, megasporangia, and microsporangia. Averagely, there were about 12 whorls for each plant, including the former 11 whorls of sporophylls and last whorl of immature leaves. We collected all whorls of sporophylls or immature leaves and sporangia, including the former 7 whorls of megasporangia and latter 4 whorls of microsporangia. Expression analysis revealed that the *IsLFY1* transcripts were slightly expressed in the former 4 whorls of megasporophylls and megasporangia, and expression levels were increasing from the 5th to 7th whorls (Fig. 4). Moreover, the expression levels in the 7th whorl of megasporangia were the highest in all collected samples. Generally, the expression levels were higher in the 8th to 11th whorls of microsporophylls and microsporangia than that from the 1th to 5th whorls, respectively. Furthermore, expression level was higher in the 11th whorl of microsporangia than the other whorls of sporangia. For the 12th whorl for immature leaves, the expression levels were relatively higher than the other whorls of sporophylls. In addition, the expression levels at roots were prominently strong. For the *IsLFY2* transcripts, expression levels in all tissues were similar with that of the *IsLFY1* transcripts (Fig. 4). But *IsLFY2* expressions were significantly higher than that of *IsLFY1* at the corms. Similarly, the expression level of *IsLFY2* was the highest in the 7th whorl of megasporangia, followed by the roots and 11th whorl of microsporangia.

### *In situ* hybridization

**Figure 5:**
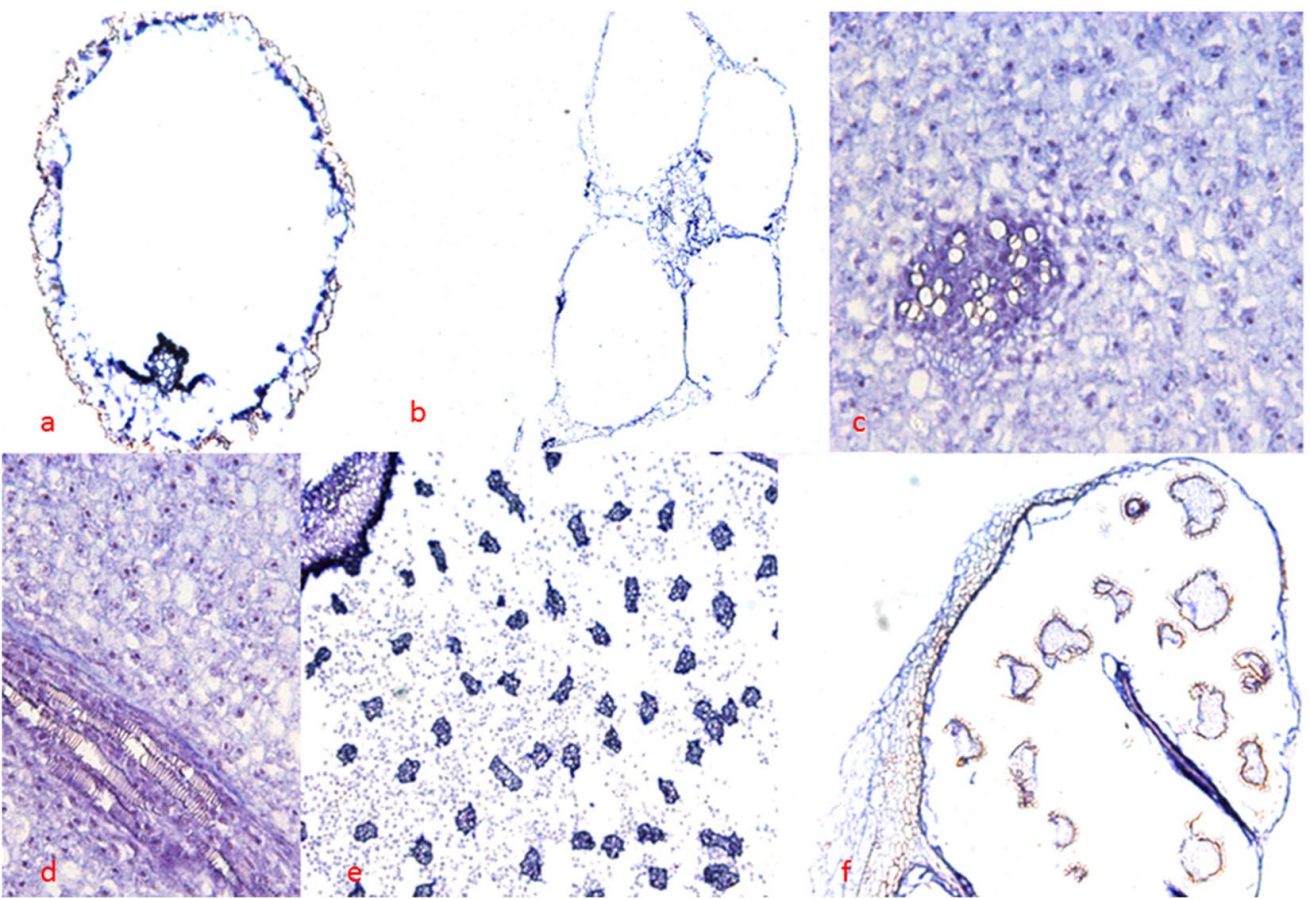
*In situ* hybridization of *IsLFY1* expressions. (**a**) Transverse section of roots. (**b**) Transverse section of leaves. (**c**) Transverse section of corms. (**d**) Longitudinal section of corms. (**e**) Transverse section of microsporangia. (**f**) Transverse section of megasporangia. Scale bars were 300 μm for megasporangia and microsporangia, 200 μm for root, leaves, and 100 μm for corms.

**Figure 6:**
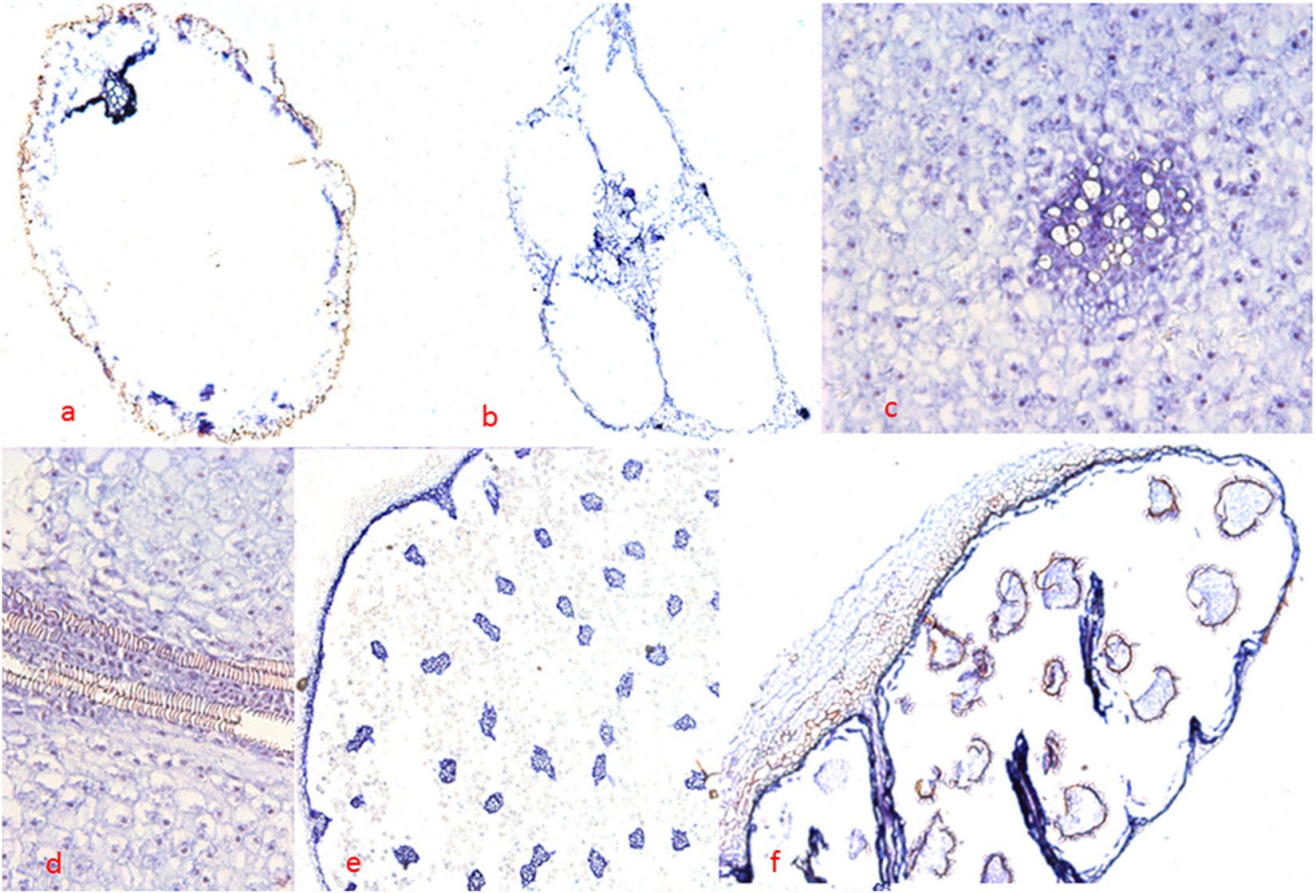
*In situ* hybridization of *IsLFY2* expressions. (**a**) Transverse section of roots. (**b**) Transverse section of leaves. (**c**) Transverse section of corms. (**d**) Longitudinal section of corms. (**e**) Transverse section of microsporangia. (**f**) Transverse section of megasporangia. Scale bars were 300 μm for megasporangia and microsporangia, 200 μm for root, leaves, and 100 μm for corms.

Expression patterns of the *IsLFY1* and *IsLFY2* transcripts were further characterized using *in situ* hybridization assays. A range of tissues were selected, containing roots, corms, and the 6th whorl of leaves and megasporangia, and 8th whorl of microsporangia. The *IsLFY1* and *IsLFY2* transcripts showed similar expression patterns in all examined tissues. The transcripts in roots were accumulated in all parts of roots except periderm (Figs 5a and 6a). Moreover, the two transcripts were significantly detected in endodermis, connective parts, and vascular bundles. Leaves of *I. sinensis* are quadrangular in outline and the two transcripts were detected all parts of the leaves, including parenchyma cells and vascular bundles (Figs 5b and 6b). Corm of *I. sinensis* is a short tuberous body^35^. *In situ* hybridization for longitudinal sections of the corms showed that the two transcripts were dramatically expressed at the zone of parenchyma cells which surrounded vascular bundles (Figs 5d and 6d). The expressions were slight in the cortical cells from transverse sections of the corm (Figs 5c and 6c). In addition, megasporangia and microsporangia are in the bottom of the sporophylls (Figure S2), and the spores are separated by trabeculae^29^. In general, a large number of microspores are located in microsporangia and limited megaspores are in megasporangia. *IsLFY1* was strongly expressed in trabeculae or intine of the microsporangia, and partial microspores (Fig. 5e). *IsLFY2* was also strongly expressed in trabeculae or intine of the microsporangia, whereas it is faintly detected in microspores (Fig. 6e). In addition, the two transcripts were expressed at high levels in trabeculae or intine of the megasporangia and expressed at a low level in megaspores (Figs 5f and 6f).

### *Arabidopsis* transgenes

**Figure 7:**
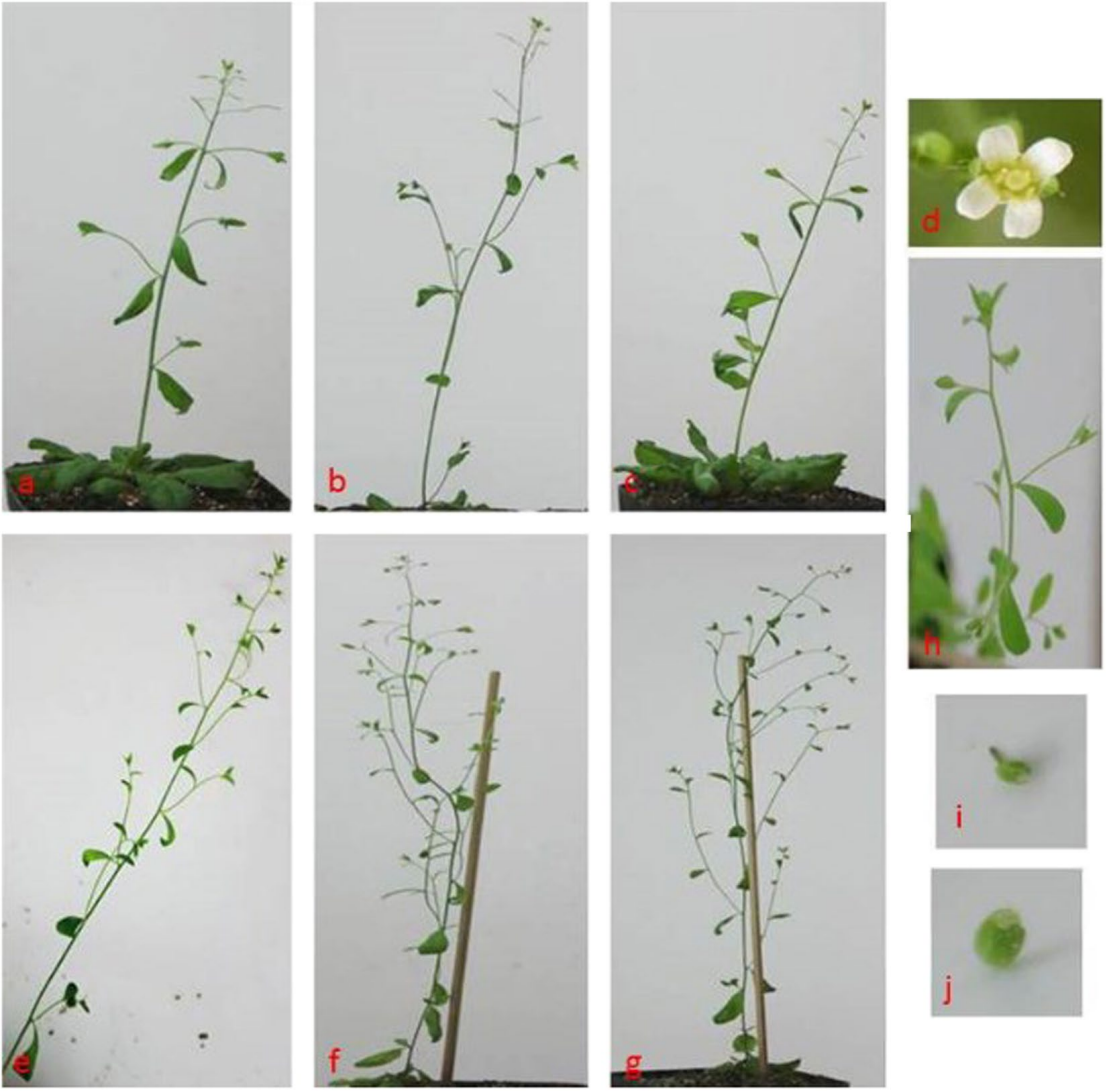
Phenotypic characters in *Arabidopsis* of constitutive *IsLFY1* and *IsLFY2* expressions. Figures from a to d are related to transgenes that the genes were crossed into wild *Arabidopsis* plants. Four-week *IsLFY1* transgenic line (**a**), wild *Arabidopsis* plants (**b**), and *IsLFY2* transgenic line (**c**). The *IsLFY1* and *IsLFY2* transgenic plants did not differ significantly in appearance from the wild-type plant. Flowers for the transgenic plants were consistent with the wild plants (**d**). Figures from e to j are related to transgenes that the genes were crossed into *lfy-1* mutants. *lfy-1* mutant (**e**). *IsLFY1* transgenic line (**f**). *IsLFY2* transgenic line (**g**). The *IsLFY1* and *IsLFY2* were inactive to rescue the *lfy* mutants. The transgenic plants and *lfy* mutant consistently showed that the early flowers were replaced by bracts, which subtended secondary inflorescences (**h**). The later flowers consisted of sepals and carpels (**i** and **j**).

To investigate whether the roles of *IsLFY1* and *IsLFY2* were conserved in evolutionary process, we created transgenic *Arabidopsis* plants, which constitutively expressed *IsLFY1* and *IsLFY2* under the control of the cauliflower mosaic virus 35S promoter, respectively. The T1 seeds were selected using kanamycin resistance and resistance plants were grown under long-day conditions. In total, we isolated 30 *IsLFY1* transgenic *Arabidopsis* lines, and only two accelerated flowering. Totally, 25 *IsLFY2* transgenic lines were identified and only one showed precocious flowering. As shown in Table S3, the remaining transgenic plants did not differ significantly in appearance from the wild-type controls and they did not cause precocious flowering (Fig. 7a–d).

In addition, we also created the transgenic plants that the full-length *IsLFY1* and *IsLFY2* cDNAs were transformed into severe *lfy-1 Arabidopsis* mutants under the control of the cauliflower mosaic virus 35S promoter, respectively. In total, we produced 6 *IsLFY1* transgenic and 5 *IsLFY2* transgenic lines (T1). The phenotypic characters were investigated from the F2 progeny. For *lfy-1* mutants, early-arising flowers are replaced by bracts and completely transformed into inflorescence shoots (Fig. 7e). The later-arising flowers are abnormal without petals and stamens^5^. Whereas wild-type flowers include four sepals, four petals, two carpels, and six stamens (Fig. 7d). Among the 200 T1 lines examined, 30 were homozygous for *lfy-1* mutants. However, only one *IsLFY1* transgenic lines partially complement the *lfy* phenotype. The remaining plants showed inactive to rescue the *lfy* mutants (Fig. 7f). In addition, among the identified 28 transgenic *lfy-1* mutants, only one *IsLFY2* transgenic lines partially complement the *lfy* phenotype and the remaining plants similarly were inactive to rescue the *lfy* mutants (Fig. 7g). The early flowers were replaced by bracts, which subtended secondary inflorescences (Fig. 7h). The later flowers consisted of sepals and carpels (Fig. 7i,j). Phenotypes of the transgenic plants were indistinguishable from the *lfy* mutants (Table S4).

## Discussion

Analysis of the deduced ILFY1 and ILFY2 protein in five *Isoetes* species showed that ILFY1 and ILFY2 amino acid sequences were common in *I. yunguiensis*, *I. sinensis*, *I. orientalis*, and *I. taiwanensis*. There was 94.86% identity for ILFY1 and 98.68% identity for ILFY2 in the four species compared with *IhLFY1* and *IhLFY2*, respectively. Moreover, the ORF fragments of *ILFY1* and *ILFY2* shared 63.41% identity for the four species and 62.57% identity for *I. hypsophila*. The five *Isoetes* species in China display a space order of the distribution pattern, *I. hypsophila-I. yunguiensis*-*I. sinensis*-*I. orientalis*-*I. taiwanensis*, from high altitude to low altitude and from west to east distribution^36, 37^. Therefore, geographic isolation probably results in a slight diversity of the *ILFY* homologs in phylogenetic evolution. *LFY* homologs possess a markedly conserved C-terminal domain and partially conserved N-terminal domain^1, 38^. Comparison analysis revealed that ILFY1 and IFLY2 in the C-terminal regions were highly conserved in the evolutionary process. The clearly conserved C-terminal regions include DNA binding domain to regulate downstream genes, whereas the partially conserved N-terminal regions play key roles in forming LFY dimerization and higher complex with other TFs or chromatin remodelers^39–41^. Moreover, An amino acid substitution from His to Asp in the C-terminal domain causes PpLFY proteins inactive by binding a canonical LFY-binding domain^38^. Nonetheless, ILFY1 and IFLY2 consistently shared the conserved His, which were resembled that in vascular plants.

Information about the expression patterns of *IsLFY1* and *IsLFY2* suggested that functions for the two genes might be conserved, probably being redundant in both paralogs, despite differences outside the DNA binding domains^17, 42^. In general, there is a single copy in most angiosperms and more than two copies in nonflowering plants. Nonetheless, major subfunctionalization is not occurred for the additional copies^20, 43^. *I. sinensis* includes sporophylls and immature leaves, and the leaves are closely spirally arranged on the fleshy corms^29^. The megasporophylls are located outside of the corm, and micriosporophylls are growing inside of the corm. Moreover, the leaves are becoming mature from inside to outside, and the immature leaves are in the center of the corm^28^. Generally speaking, the *IsLFY1* and *IsLFY2* transcripts were consistently higher in microsporangia than megasporangia, and expression levels were higher in the inside microsporophylls than outside megasporophylls. Expression levels of the two genes were similarly highest in the 7th whorl of megasporophylls, followed by roots, the 11th whorls of microsporangia, and the transcripts were highly expressed in young tissues. Thus, the results demonstrated that the two *ILFY* paralogs played general roles in both reproductive and vegetative developments. *In situ* hybridization analysis further revealed that *IsLFY1* and *IsLFY2* were ubiquitously expressed in all active parts of leaves and roots, trabeculae and intine of the megasporangia or microsporangia, and the zone of parenchyma cells which surrounded vascular bundles of the corms. The cambium appears in parenchyma cells which surrounded vascular bundles of the corms and it is not differentiated. Moreover, the transcripts were significantly detected in endodermis of the roots, which are poorly differentiated^29^. The results probably indicated that *IsLFY1* and *IsLFY2* probably have an ancestral role in controlling cell division or arrangement and developmental process^16, 21^.

Constitutive overexpression of *LFY* in *Arabidopsis* promotes early flowering and reduces number of adult leaves^4, 8^. The transgenic plants transformed lateral shoots into terminal flowers. *LFY* is strongly expressed in floral primordia, and faintly expressed in cauline leaf primordia^4^. Moreover, constitutive overexpression of *LFY* homologs results in precocious flowering in most angiosperms and gymnosperms^15, 17, 18^. However, most of the *IsLFY1* and *IsLFY2* transgenic lines did not show precocious flowering relative to the wild-type plants, suggesting that the two paralogs were not functional orthologs with *LFY* ^4, 17, 18^. The early-arising flowers in severe *lfy* mutants are converted into leaves and lateral shoots, and the late-arising flowers are only composed of sepals and carpels^4, 5, 44^. Observation revealed that the *35S::IsLFY1* and 35S::*IsLFY2* transgenes were similarly inactive to complement *lfy* mutants, which were consistent with PpLFY1 and PpLFY2 in *P. patens*. Previous research proposed that complementation ability is gradually increasing from mosses to angiosperms, and a continuum of nonneutral change is responsible for the functional changes^1^. Moreover, the gradual activity among nonflowering plants implies alteration of the DNA binding specificity of LFY homologs. It is necessary to further investigate the interaction targets of *ILFY* to elucidate the evolutionary process.

In summary, we cloned two *ILFY* paralogs in five *Isoetes* species, including *I. hypsophila*, *I. yunguiensis*, *I. sinensis*, *I. orientalis*, and *I. taiwanensis*. Expression patterns of *IsLFY1* and *IsLFY2* in *I. sinensis* demonstrated that the two genes did not have functional divergences. The two transcripts are expressed not only in reproductive tissues but also in vegetative tissues. Moreover, the transcripts were expressed at a high level in juvenile tissues, indicating *ILFY* genes control both vegetative and reproductive developments in *Isoetes*. In addition, overexpression of *IsLFY1* and *IsLFY2* in *Arabidopsis* similarly did not show precocious flowering compared with wild-type plants. Furthermore, the two paralogs were inactive to complement severe *lfy* mutant phenotypes, implying that the two homologs were not functional orthologs with *LFY*. Overall, the study provides important information for understanding the characters and functions of *LFY* homologs in *Isoetes* and evolutionary process.

## Methods

### Plant materials

Totally, five *Isoetes* species were collected in China and cultivated in a greenhouse of Wuhan University, including *I. hypsophila*, *I. yunguiensis*, *I. sinensis*, *I. orientalis*, and *I. taiwanensis*. The material *I. hypsophila* was collected from Daocheng in Sichuan Province, China (N29°29′; E100°14′); *I. yunguiensis* was collected from Hongfeng Lake in Sichuan Province, China (N26°29′; E106°58′); *I. sinensis* was collected from Xinan River in Zhejiang Province, China (29°28′N; 119°14′E); *I. orientalis* was collected from Songyang in Zhejiang Province, China (N28°47′; E119°12′); and *I. taiwanensis* was collected from Jinmen in Taiwan Province, China (N24°27 ′; E118° 23′).

### RNA isolation and cloning of *LFY* homologs

Initially, approximately 0.15 g juvenile leaves from the five *Isoetes* species were immediately sampled and frozen by liquid nitrogen before RNA extraction, respectively. Then total RNA was isolated using RNAiso Plus (Takara, Da Lian, China), according to the manufacturer’s protocols. Total RNA (6 μg) was incubated at 37 °C for 15 min to eliminate genomic DNA using 1 μL RNase-free DNase I (Promega, Madison, WI, USA). The treated total RNA was reverse transcribed into single-strand cDNA using M-MLV Reverse Transcriptase (Promega, Madison, WI, USA) and Oligo(dT)_18_-adaptor primer. Partial *ILFY* segments were amplified using specific primers (5′TTCAAATGGGAGCCCAGAATACC3′ and 5′GACATTGATGCGTGTGCTGGAT3′), which were designed according to Saiko Himi *et al.* ^20^. The obtained cDNA initially was diluted 10-fold, and used as templates for polymerase chain reactions (PCR) amplification. The reaction mixture (20 μL) contained 2.0 μL 10 × buffer with 2 mM MgCl_2_, 0.25 pmol forward and reverse primers, 0.1 mM dNTP, 2.0 μL diluted cDNA, 12.6 μL sterile water and 0.5 μL Primer Star HS DNA polymerase (Takara, Dalian, China). The PCR conditions included initial denaturation step of 5 min at 95 °C, followed by 40 cycles of 95 °C for 30 s, 55 °C for 40 s, and finally 72 °C for 1 min. Then the PCR products were purified (QIAquick PCR cleanup kit), cloned into the pEASY-Blunt vector (TransGen Biotech Company, Beijing, China), and sequenced in both directions on an ABI 3730 DNA Sequencer using BigDye Terminator version 3.1 (Applied Biosystems).

To obtain full-length ORF of *ILFY* sequences in the five *Isoetes* species, we performed 5′ and 3′ RACE PCR using the 5′ RACE System for Rapid Amplification of cDNA Ends and 3′ RACE System for Rapid Amplification of cDNA Ends (Invitrogen™) Kits, respectively. The gene-specific primers for the 5′ RACE PCR were included (5′GATCTTTGCCTTTCCGTCTCATCCTCC3′ and (5′CCAAGCTCTGCAACCTTTGCTATTGTGC3′). The gene-specific primers for the 3′ RACE PCR were used (5′GGTCCCAGGGGATATGAATGGCAGAG3′ and 5′TGGAGGATGAGACGGAAAGGCAAAGA3′). Then the products for the 5′ and 3′ RACE PCR were purified and cloned into pEASY-Blunt Cloning vector, respectively. Eight positive clones were sequenced in both directions for each sample.

### DNA isolation, amplification and sequencing

Total genomic DNA of the five *Isoetes* species was isolated from juvenile leaves using the CTAB method^45^. The two *ILFY* paralogs were amplified using specific primers (*ILFY1*: 5′ATGACTGAGCTGGGTTTCACCG3′ and 5′TTAGCTGCTTTCTTTGCTTTTTTCT3′; and *ILFY2*: 5′TCAAATGGGAGCCCAGA3′ and 5′TTAGCTGCACTCTTTACCCTT3′) respectively. The PCR program was an initial denaturation step of 5 min at 95 °C, followed by 30 cycles of 95 °C for 30 s, 57 °C for 50 s, and finally 72 °C for 2 min. The products were further purified and ligated with pEASY-Blunt Cloning vector. Three positive clones for each sample were sequenced in both directions for each sample.

### Construction of a phylogenetic tree

LFY homologs were searched using the program BLAST X model in the NCBI database (http://www.ncbi.nlm.nih.gov/). The amino acid and nucleotide sequences were aligned using Clustal X 2.0 software^46^, including 36 *LFY* homologous sequences and 10 *ILFY* homologous sequences obtained in this study. The phylogenetic analysis was conducted based on the JTT substitution model and maximum likelihood approach implemented in the Molecular Evolutionary Genetics Analysis version 7.0 (MEGA 7.0) program^47^. Support for internal nodes was estimated based on 1000 bootstrap replicates, and bootstrap less than 50% was removed above the relevant branch^48^.

### qRT-PCR

Given that *I. sinensis* is relatively widespread in China and easily adapts to the greenhouse environment in Wuhan University, *I. sinensis* was employed to investigate the expression patterns, including *IsLFY1* and *IsLFY2*. The leaves and sporangia were arranged on corms in whorls, and they are becoming mature from inside to outside^29^. Each whorl of green leaves and sporangia was collected, respectively. All sampled materials were immediately frozen by liquid nitrogen for RNA extraction. In addition, we also sampled the materials of corms and roots for RNA isolation. Total RNA of all samples was isolated using RNAiso^TM^ Plus (Takara, Da Lian, China). Then the isolated RNA was treated with RNase-free DNase I (Takara, Da Lian, China) for 45 min according to the manufacturer's protocols. Subsequently, 1 μg treated RNA was reverse-transcribed into single-strand cDNA using Primerscript^TM^ One Step RT-PCR Kit Ver.2 (Takara, Da Lian, China). The qRT-PCR experiments for *IsLFY1* and *IsLFY2* were carried out on a CFX96 Real-time PCR system (Bio-Rad, Hercules, USA) with *IsLFY1*-specific primers (5′GATGCTCTTGATGTGCAAGC3′ and 5′TCACCACACAGATTGACTC3′) and *IsLFY2*-specific primers (5′GAAAGACCACCAAGGAAA3′ and 5′AAGCAGACAGTCGGAAAG3′), respectively. The first-strand cDNA initially was diluted 10-fold and used as templates in the qRT-PCR tests. The reaction mixture (25 μL) included 0.25 pmol forward and reverse primers, 12.5 μL 2 × SYBR premix (Takara, Da Lian, China), 2.5 μL diluted cDNA, and 7.0 μL sterile water. The thermocycling condition was an initial denaturation step of 5 min at 95 °C, followed by 40 cycles of 95 °C for 30 s, 60 °C for 15 s, and finally 72 °C for 30 s. A melting curve was plotted to check the specific products of amplification reactions at the end of the PCR cycling over the range 65–95 °C. Baseline and threshold cycle (Ct) were determined automatically by the Bio-Rad CFX Manager 2.1 software. The relative expression levels were calculated using 2^−△△Ct^ method, and normalized to the geometric average of Ct values with *Actin* as an internal control^24, 49^. All experiments and analyses were performed in triplicate.

### *In situ* hybridization

*I. sinensis* was further employed to investigate spatial-specific expression patterns of *IsLFY1* and *IsLFY2* using in situ hybridization assays. Samples were fixed using 4% paraformaldehyde buffers, including roots, corms, and the 6th whorl of sporophylls and megasporangia, and 8th whorl of microsporangia. In order to ensure the specific hybridization patterns of *IsLFY1* and *IsLFY2*, the non-conserved regions of *IsLFY1* and *IsLFY2* were used to design the specific probes. Partial sequences of *IsLFY1* were amplified using *IsLFY1*-specific primers (5′AATCTATGCTGGCTACTGG3′ and 5′TTGAGTCGCACGTCGTAT3′); and partial sequences of *IsLFY2* were amplified using *IsLFY2*-specific primers (5′GGACTATGGTGTTAGGCTCT3′ and 5′CAGATGTCCCTGTATTTGC3′). The two amplification segments were introduced into the pGEM-T Vector (Promega, Madison, WI, USA), respectively. Antisense probes for the two paralogs were synthesized using *Sac*II (Takara, Da Lian, China) and SP6 RNA polymerases (Promega, Madison, WI, USA). Sense strand controls for the *IsLFY1* and *IsLFY2* probes were synthesized using *Sac*I (Takara, Da Lian, China) and T7 RNA polymerases (Promega, Madison, WI, USA). Both the sense and antisense probes of *IsLFY1* and *IsLFY2* were generated into single-stranded digoxigenin-11-UTP-labeled RNA probes, respectively. Tissues materials were fixed, embedded with paraplast, sectioned into 8 μM in thickness, and hybridized using the probes, described by Bechtold *et al*.^50^. All slides were photographed using an OLYMPUS X73 microscope.

### Plasmids construction and *Arabidopsis* transgenes

The coding regions of *IsLFY1* and *IsLFY2* were ligated with the binary vector pBI121 with a cauliflower mosaic virus 35S promoter, respectively. Initially, *Bam*HI and *Sma*I restriction fragments were added to the upstream of start codon sequences and downstream of stop codon sequences for the two gene*s*, respectively. The introduced restriction fragments of *IsLFY1* and *IsLFY2* were generated by PCR amplification using a set of primers (*IsLFY1*: 5′CGGGATCCATGACTGAGCTGGGTTTCACCG3′ and 5′TCCCCCGGGTTAGCTGCTTTCTTTGCTTTTTTCT3′; and *IsLFY2*: 5′CGGGATCCATGACAGATTCAGAGATAGAAGAAC3′ and 5′TCCCCCGGGTTAGCTGCACTCTTTACCCTT3′). The amplified fragments were digested with *Bam*HI and *Sma*I, and further cloned into the *Bam*HI-*Sma*I sites of pBI121 binary vector. Then the constructed 35S::*IsLFY1* and 35S::*IsLFY2* were sequenced to identify the correct orientation.

The identified 35S::*IsLFY1* and 35S::*IsLFY2* were introduced into *Agrobacterium tumefaciens* strain GV3101, and further crossed into the *Arabidopsis* plants (Columbia ecotype) using the floral dip method^51^. In addition, the 35S::*IsLFY1* and 35S::*IsLFY2* were also crossed into *lfy* heterozygous *Arabidopsis* plants, respectively. The heterozygous lines were isolated from a bulk of *lfy-1* mutant seeds, which were obtained from TAIR (http://www.arabidopsis.org/). All transgenic plants (T1) were selected on MS medium containing 50 mg/mL kanamycin. The 35 S::*IsLFY1* and 35 S::*IsLFY2* lines were identified using PCR amplifications through plating 200 F1 seeds, and homozygous lines (T2) were selected for phenotypic analysis. The seeds were kept for 3 days at 4 °C before sowing. All resistant and wild plants were grown at 23 °C under 16 h light/8 h dark.

## Electronic supplementary material

Supplementary Information
