# Supplementary material for: Two LEAFY homologs ILFY1 and ILFY2 control reproductive and vegetative developments in Isoetes L
Source: Sci Rep. 2017 Mar 22;7:225. doi: 10.1038/s41598-017-00297-3 (PMC5412651; doi:10.1038/s41598-017-00297-3)
Supplement: Supplementary file 1 — Supplementary Information [file 41598_2017_297_MOESM1_ESM.pdf]

Two *LEAFY* homologs *ILFY1* and *ILFY2* control reproductive and vegetative developments in *Isoetes* L.

Tao Yang, Ming-fang Du, You-hao Guo*, Xing Liu *

Laboratory of Plant Systematics and Evolutionary Biology, College of Life Science, Wuhan University, Wuhan, Hubei, China.

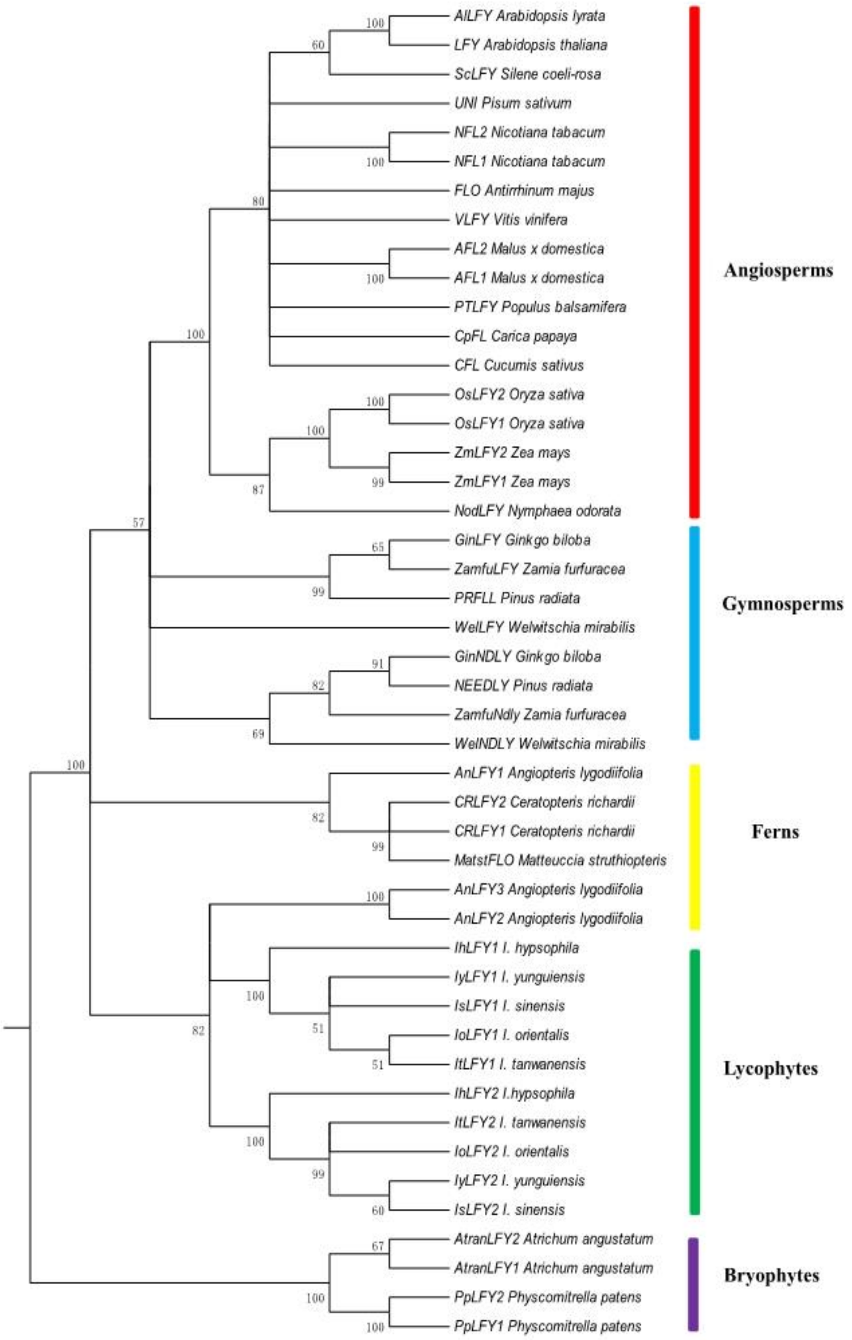

Figure S1. Phylogenetic tree of *LFY* homologs.

The phylogenetic tree was constructed using the conserved N-terminal and C-terminal regions of the *LFY* nucleotide sequences. Numbers above the branches represent

bootstrap value, and bootstrap less than 50% was removed. Accession numbers for the *LFY* homologs in the dataset were listed in Table S5.

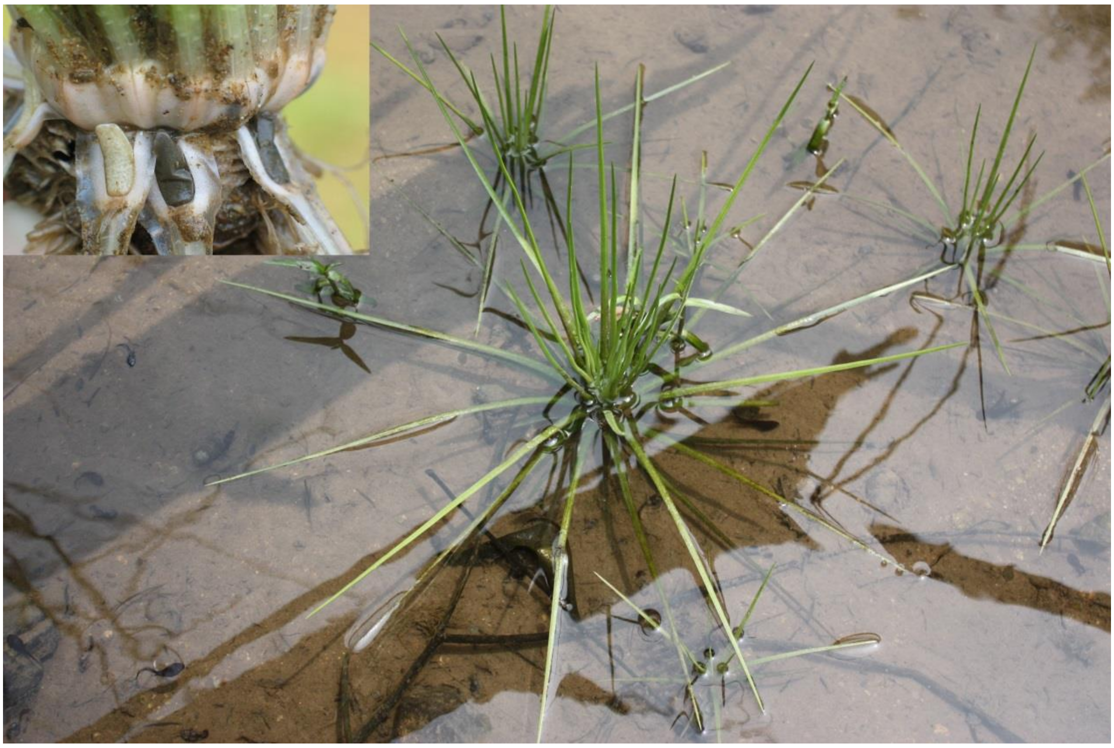

Figure S2. Morphologic profile of *Isoetes sinensis*.

The picture in the top left presents the structure of megasporangia and microsporangia. The white sporangium is the immature megasporangium and the black sporangium is the mature microsporangium.

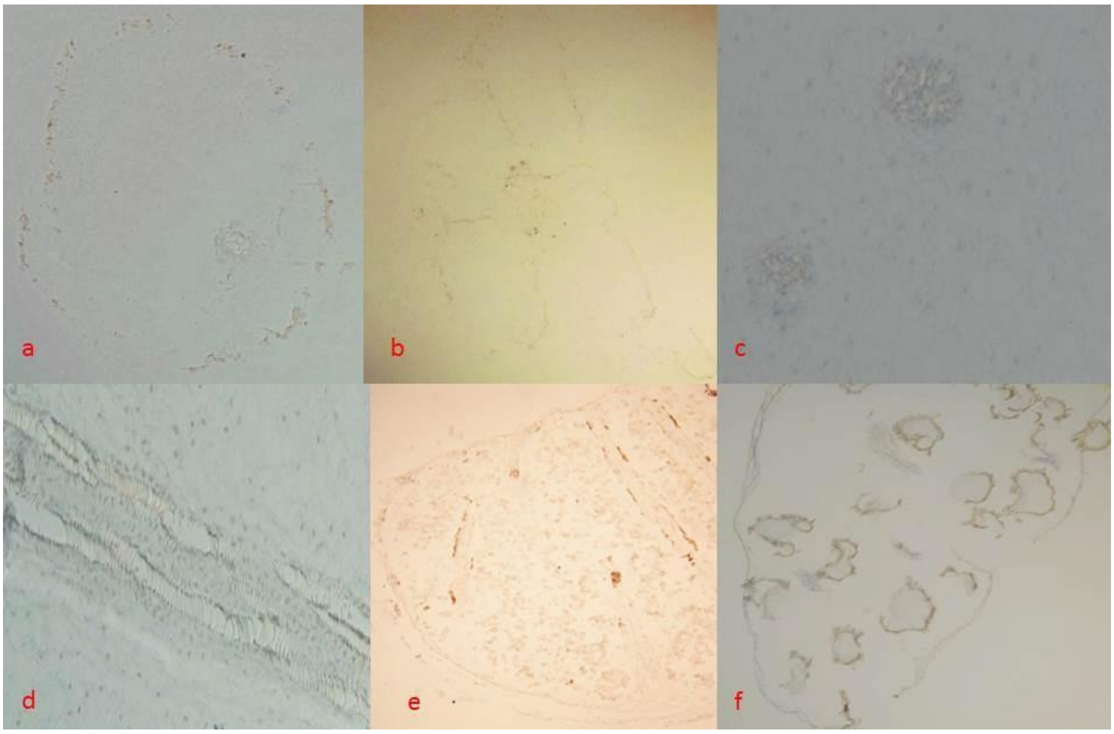

Figure S3. Negative control of *IsLFY1*expressions in in situ hybridization assays.

a) Transverse section of roots. b) Transverse section of leaves. c) Transverse section of corms. d) Longitudinal section of corms. e) Transverse section of microsporangia. f) Transverse section of megasporangia. Scale bars were 300 μm for megasporangia and microsporangia, 200 μm for root, leaves, and 100 μm for corms.

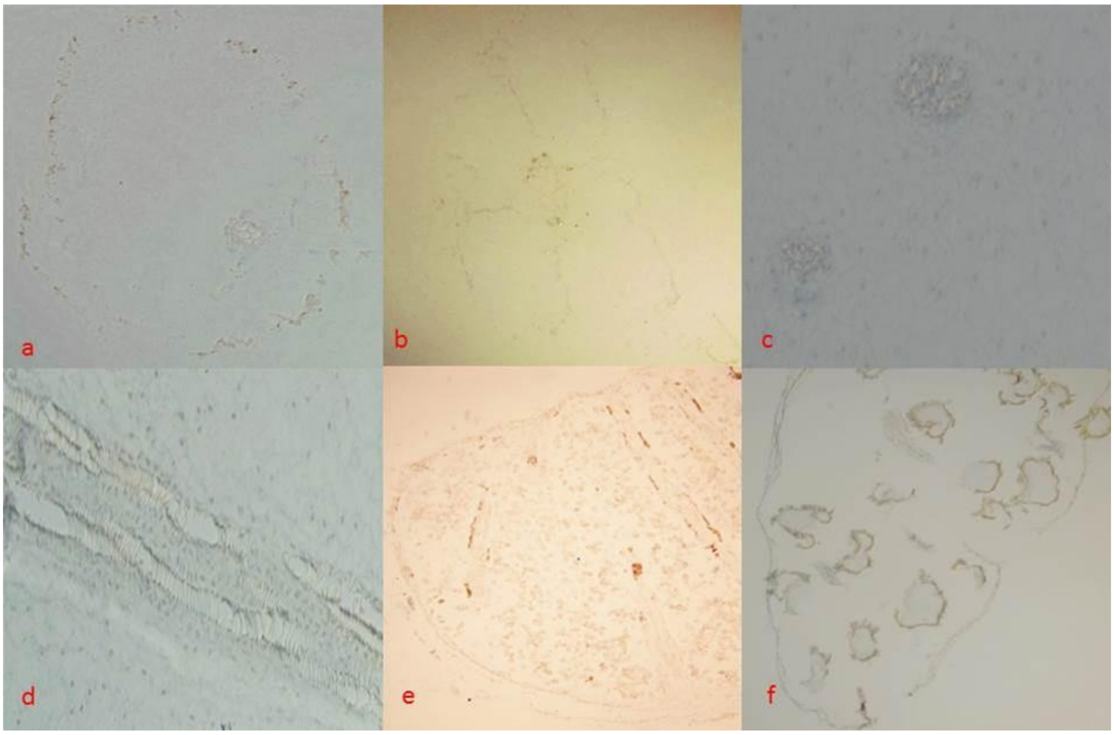

Figure S4. Negative control of *IsLFY2*expressions in in situ hybridization assays.

a) Transverse section of roots. b) Transverse section of leaves. c) Transverse section of corms. d) Longitudinal section of corms. e) Transverse section of microsporangia. f) Transverse section of megasporangia. Scale bars were 300 μm for megasporangia and microsporangia, 200 μm for root, leaves, and 100 μm for corms.

**Genes**	**Full length cDNA (bp)**	**5'UTR (bp)**	**ORF (bp)**	**3' UTR (bp)**
IhLFY1	1449	33	927	489
IyLFY1	1458	33	936	489
IsLFY1	1458	33	936	489
IoLFY1	1458	33	936	489
ItLFY1	1458	33	936	489
IhLFY2	1768	744	726	298
IyLFY2	1768	744	726	298
IsLFY2	1768	744	726	298
IoLFY2	1768	744	726	298
ItLFY2	1768	744	726	298

Table S1. Detailed information about *ILFY1* and *ILFY2* sequences of the five *Isoetes* species.

**Genes**	***IsLFY1***	***IsLFY2***	***IhLFY1***	***IhLFY2***
LFY	41.47%	35.71%	40.86%	36.67%
PRLFF	48.42%	43.69%	48.06%	43.69%
NEEDLY	51.49%	46.53%	51.24%	46.53%
CRLFY2	52.03%	46.45%	52.03%	46.45%
SmLFY1	46.20%	43.17%	45.55%	43.17%
PpILFY1	50.00%	43.82%	49.32%	43.82%

Table S2. Identical levels of amino acid sequences compared deduced proteins of IhLFY1, IhLFY2, IsLFY1,and IsLFY2 with LFY of *Arabidopsis thaliana*, PRLFF and NEEDLY of *Pinus radiata*, CRLFY2 of *Ceratopteris richardii*, SmLFY1 of *Selaginella moellendorffii*, and PpILFY1 of *Physcomitrella patens*, respectively.

	Days from sowing to flowering	Number of rosette leaves	Number of plants
Wild type(Col)	30 ±1.4	11 ±1.2	40
35S::*IsLFY1*	32 ±0.8	10 ±1.8	30
35S::*IsLFY2*	29 ± 2.2	12 ±0.5	25

Table S3. Characters of wild-type *Arabidopsis* and transgenic plants.

	Number of rosette leaves	Number of cauline leaves	Number of secondary inflorescence	Number of plants
Wild type(Col)	11 ±1.2	3.9±0.2	2.9±0.2	40
lfy-1	11.5 ±2.2	10±1.1	21.5±0.5	10
35S::*IsLFY1*	11.8 ±1.8	12±1.1	19.5±1.2	30
35S::*IsLFY2*	12.5 ±0.4	10.1±2.1	22.8±0.8	28

Table S4. Phenotype of wild-type plants, *lfy-1* mutant and transgenic plants.

**Species**	**LEAFY homologs**	**Accessions for proteins**	**Accessions for cDNA**
Ceratopteris richardii	*CRLFY1*	BAB41069.2	AB049974.2
Ceratopteris richardii	*CRLFY2*	BAB41070.2	AB049975.2
Ginkgo biloba	*GinLFY*	AAF77075.1	AF108228.1
Ginkgo biloba	*GinNDLY*	AAF77074.1	AF105111.1
Pinus radiata	*NEEDLY*	AAB68601.1	U76757.1
Pinus radiata	*PRFLL*	O04116	U92008.1
Nicotiana tabacum	*NFL1*	Q40504	NM_001326104.1
Nicotiana tabacum	*NFL2*	Q40505	AH006599.2
Physcomitrella patens	*PpLFY1*	BAB60676.1	AB052251.1
Physcomitrella patens	*PpLFY2*	BAB60677.1	AB052252.1
Welwitschia mirabilis	*WelLFY*	AAF23870.1	AF109130.1
Welwitschia mirabilis	*WelNDLY*	AAD41615.1	AF072369.1
Zamia furfuracea	*ZamfuLFY*	AAF77606.1	AF105107.1
Zamia furfuracea	*ZamfuNdly*	AAF77607.1	AF105108.1
Malus x domestica	*AFL1*	BAB83096	AB056158.1
Malus x domestica	*AFL2*	BAB83097	AB056159.1
Angiopteris lygodiifolia	*AnlLFY1*	BAB88867.1	AB050090.1
Angiopteris lygodiifolia	*AnlLFY2*	BAB88868.1	AB050091.1
Angiopteris lygodiifolia	*AnlLFY3*	BAB88869.1	AB050092.1
Atrichum angustatum	*AtranLFY1*	AAG42694	AF286054.1
Atrichum angustatum	*AtranLFY2*	AAG42695	AF286055.1

Cucumis sativus	*CFL*	AAC64705.1	AF059320.1
Antirrhinum majus	*FLO*	AAA62574.1	M55525.1
Silene coeli-rosa	*ScLFY*	CAC86163	AJ311804.1
Pisum sativum	*UNI*	AAB88139.1	AF035163.1
Oryza sativa	*OsLFY1*	XP_015635355	XM_015779869.1
Oryza sativa	*OsLFY2*	Q0JAI1	KF051023.1
Nymphaea odorata	*NodLFY*	AAF77609.1	AF105110.1
Matteuccia struthiopteris	*MatstFLO*	AAF77608.1	AF105109.1
Zea mays	*ZmLFY1*	NP_001105201	NM_001111731.1
Zea mays	*ZmLFY2*	AAV68213	AY789034.1
Arabidopsis thaliana	*LFY*	AAA32826	M91208.1
Arabidopsis lyrata	*AlLFY*	AAM27942.1	JQ180307
Carica papaya	*CpLFY*	AAY57438	DQ054794.1
Populus balsamifera	*PTLFY*	AAB51533.1	U93196.1
Vitis vinifera	*VLFY*	AAN14527.1	AF450278.1

Table S5. Accession numbers related to the protein and nucleotide sequences of *LFY* homologs used in this study.
